# Future‐Proofing Dentistry: A Qualitative Exploration of COVID‐19 Responses in UK Dental Schools

**DOI:** 10.1111/eje.13055

**Published:** 2024-11-19

**Authors:** Jon J. Vernon, Karen Vinall‐Collier, Julia Csikar, George Emms, Paula E. Lancaster, Brian R. Nattress, David J. Wood

**Affiliations:** ^1^ Division of Oral Biology, School of Dentistry University of Leeds Leeds UK; ^2^ Department of Dental Public Health, School of Dentistry University of Leeds Leeds UK; ^3^ School of Dentistry University of Leeds Leeds UK; ^4^ Division of Restorative Dentistry, School of Dentistry University of Leeds Leeds UK

**Keywords:** aerosols, COVID‐19, dentistry, education, SARS‐CoV‐2, ventilation

## Abstract

**Introduction:**

The COVID‐19 pandemic had extensive influence on dental education. UK dental schools were compelled to respond with substantial adaptations to clinical training approaches and environments to mitigate educational impact.

**Materials and Methods:**

The Surveying Pandemic Education Response in Higher Education Dental Schools (SPEARHEAD) study aimed to retrospectively evaluate the diverse responses of UK dental schools to the COVID‐19 pandemic. All UK dental schools were invited to participate in semi‐structured interviews to ascertain institutional responses, with transcripts subjected to thematic framework analysis.

**Results and Discussion:**

Ten UK dental schools contributed to the study and three main themes were identified: student education, environment, and procedures and equipment. The most common approach to student education was the reduction of student numbers in clinical areas; however, this increased supervisory demands. While there was widespread acknowledgement of the need for enhanced ventilation, implementing the necessary modifications was frequently constrained by building configurations and financial implications. Numerous procedural adjustments were implemented, accompanied by widespread adoption of enhanced personal protective equipment. Fallow periods were common, although differing durations underscored the need for data‐driven guidance. Many schools transitioned towards electric speed‐controlled handpieces, but the need to reflect real‐world scenarios often led to a reversion to air turbines.

**Conclusion:**

UK dental schools showed initiative, resilience, and ingenuity in safeguarding students from enduring irretrievable educational setbacks amidst the challenges posed by the COVID‐19 pandemic. Validating a data‐driven strategy for addressing future threats would facilitate a unified response, minimising the educational repercussions and bolstering the resilience of dental training.

## 1 Introduction

On 25 March 2020, the UK government ordered a nationwide lockdown in response to the COVID‐19 pandemic. This included all non‐urgent dental services and educational facilities [1, 2, 3], leaving UK dental schools desperately seeking strategies for the safe reintroduction of clinical teaching activities for large cohorts of dental students. Due to the many uncertainties surrounding the virus and its modes of transmission [4], infection prevention and control guidance was slow to emerge. Furthermore, when recommendations for mitigation strategies were communicated, they were often based on minimal empirical research data [5].

The common conception was that COVID‐19 was transmitted primarily through respiratory routes [4], which underlined the concern surrounding dentistry. Since dental aerosol‐generating procedures (AGPs) cover a range of clinical activities [6], this highlighted the need for decision‐making to be guided by aerosol research to identify optimum mitigation strategies to minimise the risk of viral aerosol dissemination. As the pandemic persisted, valuable research arose regarding the use of electric speed‐controlled handpieces (with the air coolant turned off) or the placement of rubber dams to minimise the risk from viral aerosols [7, 8, 9, 10, 11, 12]. However, by then many operational decisions were already made, such as introducing electric handpieces with reduced cutting speeds of 60 000 rpm and fallow periods, as the necessity to provide clinical opportunities for dental students, particularly final years, was clear. Ensuring students developed the clinical competencies required as “safe beginners/practitioners” was of utmost importance, and in two Scottish Dental Schools (Dundee and Glasgow) a 1‐year extension was required to reach this status [2, 13]. Since many of the restrictions have eased and the imminent outbreak threat has subsided, the majority of services have returned to business as usual. Nonetheless, dental settings must continue to adapt to the threat of future pandemics with regards to aerosol reduction approaches, whether through new recommended equipment, procedures, personal protective equipment (PPE) or improved ventilation strategies.

Here, we sought to obtain first‐hand accounts of UK dental school responses at different stages of the pandemic through interviews with key stakeholders. By assessing different approaches to the pandemic response and identifying commonalities, we seek to gather a greater understanding of the issues raised. By focusing on dental education facilities, we believe that the most innovative approaches that may have been less constrained by the burden of NHS demands can be recognised and potentially translated to improving public and private practices. Furthermore, by gaining a deeper understanding of barriers to mitigating actions and retaining any lessons learnt, we aim to identify strategies for the future‐proofing of dental education to address any potential challenges.

## 2 Aim

This study aims to provide a retrospective evaluation of the changes made in UK dental schools in response to COVID‐19 and the barriers to their implementation.

## 3 Materials and Methods

### 3.1 Setting, Participants and Recruitment

All UK dental schools (*n* = 16) were approached via the Dental Schools Council (DSC) to participate in the study. The Dean (or nominated senior member of staff with relevant knowledge and experience) of each dental school was invited to participate in a short, semi‐structured interview conducted over an online platform. The DSC was approached initially via email, the research team gaining permission to present the research plan and extend an invitation to each school; further details were subsequently disseminated through email via the DSC secretariat.

### 3.2 Data Collection

A topic guide was developed from the existing literature on dental aerosols and pandemic response, as well as discussion with experts in the area. A semi‐structured interview style was employed with adaptable lines of questioning encouraged to elicit comprehensive insights into institutional responses. In line with iterative approach, the topic guide was modified to remove irrelevant questions and/or repetition of questions depending on previous answers given. Prior to commencing the interview, verbal consent was obtained. Interviews were conducted by a dental core trainee and were recorded and then audio‐transcribed verbatim by the video conferencing software. Transcripts were verified for accuracy (by the interviewer) prior to analysis.

### 3.3 Data Analysis

An interpretivist phenomenological approach was used to analyse the data utilising a thematic analysis based on the Framework approach [14]. Analysis focused on elucidating dental schools' response to the pandemic, barriers and drivers to mitigating actions, and lessons learnt for future‐proofing. Microsoft Excel was used to manage the interview data. The following steps were undertaken. An independent research company worked alongside the data analysis team to complete steps 1–3. Step 4 was done by the data analysis team.

1. Familiarisation: Two transcripts were read to record emerging ideas and recurrent themes that were relevant to the study aims.
2. Constructing a thematic framework: A thematic framework was drafted, structured by the topic guide, as well as ideas and themes from the previous step. The framework was piloted using a third transcript and refinements were agreed.
3. Indexing and Charting: The thematic framework was then systematically applied to the interview data. Charts were produced in Excel for each theme and summaries of responses from participants and verbatim quotes were entered.
4. Mapping and Interpretation: The completed charts were reviewed and interrogated by the data analysis team to compare and contrast views, seek patterns, connections and explanations within the data. Descriptive findings were written for each theme, then reviewed by the entire research team.

### 3.4 Ethical Considerations

The primary ethical concern was that responses may contain commercially or reputationally sensitive information and therefore, all data was pseudo‐anonymised, analysed collectively and no findings were attributed to any single school.

## 4 Results and Discussion

Interviews were conducted between May and December 2023 with representatives of 10 UK dental schools. The participants encompassed the roles of Dean of School, Director of Clinical Dentistry, Head of Education, Professor, Senior Clinical Lecturer and Head of School. Interviews lasted between 24 and 49 min. Thematic analysis enabled three key themes to be identified: student education, environment, and procedures & equipment.

### 4.1 Student Education

Universally across all dental schools reducing the number of students in the clinic was seen as one of the most effective strategies, yet one of the most complex to implement logistically and then ultimately undo once COVID‐19 precautions were reduced.

When clinical activities resumed, priority was commonly given to higher‐year students for in‐person clinical experiences. Lower‐year students often engaged in simulation exercises while waiting for an opportunity for clinical rotations. This approach allowed dental schools to manage student rotations effectively while ensuring all students received necessary hands‐on experiences.

Half‐year groups were implemented to control the flow of people in common areas. To combat the accompanying reduction in direct treatment experiences, the number of clinical sessions per day was commonly increased from two to three, often with an additional evening session.

“So, we were running as we do now, a morning clinic of four hours and an afternoon clinic of three hours. But then we also put on… evening activity, which started at 5:15 pm and finished at 7:30 pm.” [PARTICIPANT 7]

A shift towards reduced student numbers in clinic did allow for a more favourable student‐to‐staff ratio, potentially improving the supervision and learning experiences for students. One‐to‐one supervision was implemented in some instances; however, it quickly became apparent that this was not feasible in the long term due to limited staff resources. Protocols were then adapted so that staff could move between bays/pods, increasing student‐to‐staff ratios closer to pre‐pandemic levels. This greater demand on supervisory time highlighted practical constraints of staffing difficulties, on top of the additional burden of losing staff time to isolation protocols and caring responsibilities.

Additionally, there was a focus on practical solutions to ensure continuity of clinical education, such as scheduling students to assist one another on specific days, creating partnerships to ensure continuity of patient care in case of illness, and implementing a system where students could step in for each other without cancelling appointments. This peer‐support system allowed for seamless patient care despite limited physical presence in clinics.

Simulation exercises were strategically integrated into the curriculum to provide students with a well‐rounded and comprehensive learning experience, ensuring they were adequately prepared for real‐world clinical practice. Simulation‐based teaching was used to ensure the maintenance of students' practical skills while AGP opportunities were limited. Schools were cautious that simulations were not a substitution for actual patient procedures, but as a valuable adjunct for students to retain hand skills. In four locations, participants described using dental manikin (phantom head) simulations, serving as a crucial preparatory step enabling students to increase in confidence for the transition back to clinical skills, if not patient interactions.

To counter the issue of reduced patient contact, two locations reported that teaching approaches were adapted by incorporating case‐based simulations. New scenarios and cases were created to cover various aspects of actual consultations, treatment planning, and different procedures. These innovations served as effective teaching tools to continue students' education when patient contact time was restricted. In one location, simulated teaching was not feasible due to the limitations of using alternative chairs in the first year of the pandemic:

“We actually didn't have enough chairs just to run the courses that we needed to run… we had to cram a load of stuff into clinical skills and had limited space because of the alternate chair use… We didn't ever swap out clinical experience for simulated experience.” (PARTICIPANT 10)

In some instances, even as clinical activities resumed, clinical scenarios developed during the pandemic were retained and integrated into simulation exercises, as their educational value was recognised by both staff and students:

“We were adamant that we weren't going to accept simulation in lieu of actual treatment on patients…. we did do additional simulation, but we really used that during the period where our AGP pods were being built… so that they would retain their hand skills… So they did do more simulation, but this was on top of their normal clinical requirements.” [PARTICIPANT 6]

There was a significant shift to online teaching methods, incorporating online meetings, lectures, and seminars. Schools took advantage of the disruption caused by the pandemic to undergo curriculum reviews and modifications, seizing the opportunity to rearrange teaching modules and implement new curricula:

“We undertook a curriculum review of our course, which was planned pre‐COVID. But actually because of the disruption to teaching and modules during COVID, it became an opportune moment to actually say well, look, let's seize the moment. Everything was being disrupted anyway, so we actually used the opportunity.” [PARTICIPANT 3]

Although a lot of online lecturing has returned to in‐person teaching, a hybrid of the two is now commonly used, where suitable. The development and improvement of online learning platforms and the constant availability of learning materials electronically represent a positive and progressive step. Student monitoring meetings, which were previously conducted face‐to‐face, have often shifted to virtual platforms. This change reflects the widespread adoption of online communication tools for educational purposes. Traditional face‐to‐face lectures have been reduced, and novel pedagogies such as flipped classrooms, where students are encouraged to engage with online materials independently and then participate in discussions, case‐based sessions, feedback sessions, and question sessions during class time, have been retained. This demonstrates educational evolution towards approaches that promote active and discussion‐based learning:

“We're not trying to get back to where we were pre‐pandemic because education has shifted… we're trying to… implement a different type of education, which is about having a better clinical teaching experience that is better supported by other methods, including simulation.” [PARTICIPANT 5]

Curriculum and pedagogical changes were essential to ensure the continuity of UK dental schools, and this aligned with US dental schools, where 71.4% reported curricula amendments [15]. Although one study reported that 97% of UK dental schools believed that their clinical skills were affected [16], it is encouraging to see evidence that all trainees in two Welsh cohorts fulfilled all the necessary elements to complete the UK dental foundation training portfolio [17], demonstrating no ongoing impediment to clinical skills.

### 4.2 Environment

The necessity for improved ventilation and airflow control was evident in the early stages of the pandemic but not always immediately achievable. Nonetheless, a variety of different adaptations were made to clinical environments to mitigate against transmission. One of the most common adaptations made was the installation of transparent plastic screens to divide clinical spaces, as these were a quick and affordable option to create physical barriers. Nonetheless, each school's clinics have unique features, with all having open‐plan layouts and some utilising single‐bay surgeries either within the dental school or in another facility. In open‐plan scenarios, a staggered approach to chair usage was often adopted, with one chair in use while surrounding chairs were left empty. This ‘checkerboard pattern’ delineated individual zones, ensuring a safe distance between dental units:

“Rather than a sort of every dental chair being used, we were operating in a kind of ‘checkerboard pattern’. So, every other chair was essentially ‘fallow’ and we had tape marked out on the floors to try and create essentially little pods around each of the individual chairs where you'd have heavily contaminated zones and you know, areas where you might still put an FFP3 on walking up and down the corridor.” [PARTICIPANT 7]

In some instances, the need for spacing between chairs was eliminated due to the use of electric handpieces operating at or below 60 000 rpm, and patients responded negatively to all COVID‐19 screening questions. Additionally, where the orientation of the chairs and the airflow within individual bays permitted, all clinic bays were used for AGPs, eliminating the need to stagger their use. Dividing open plan spaces was deemed necessary, as there was a shortage of single‐chair surgeries. This lack of segregated clinics led to some schools utilising fallow space in community dental services, which had subsequent impacts on their services. In some instances, outreach centres were setup as open‐plan clinics, with funding for conversion of spaces secured from the government. However, this was limited.

An increased awareness of ventilation systems was developed, with efforts made to assess air changes per hour (ACH) in clinical spaces. Cross‐ventilation was used in spaces with windows to act as natural ventilation and mechanical ventilation systems increased and upgraded to achieve the guideline 10 ACH [18], where possible. While efforts were made to address ventilation concerns, there was a recognition of varying air exchange rates and challenges in obtaining detailed information about ventilation in larger clinic spaces:

“Lots of lack of information, lots of uncertainty as to what our surgeries were capable of doing. And a real absence of information about the open‐plan clinics where all the undergraduate teaching was carried out.” [PARTICIPANT 5]

Therefore, schools employed a combination of natural ventilation, mechanical systems, and increased ventilation cycles to enhance air circulation and minimise the risk of aerosol transmission. Two institutions constructed self‐contained pods with enhanced ventilation to increase the number of procedures that could be performed in a single session. The pods were designed to achieve at least 10 ACH to ensure adequate ventilation and reduce the risk of aerosol transmission. However, these required significant space, finances, and effort to implement:

“Over the course of the following year, basically, we built little ‘portacabin type’… we call them ‘pods’… it's really… we'd looked at tents or like, field hospital tents stuff and actually we did build 2 tents in one of our outreach centres… we ended up building 14 [AGP] pods on our restorative main clinic… this was a staggered program because obviously this was a big undertaking.” [PARTICIPANT 6]

Nonetheless, where investments were made into building renovations and the ventilation infrastructure, commitments have been maintained. In many institutions, continued efforts are being made to monitor and improve air quality in clinical settings. High screens separating bays in open clinics have frequently been preserved. These efforts demonstrate a proactive approach to addressing ventilation concerns highlighted by others [19, 20, 21], and ensuring a safer environment for both staff and patients in the dental clinics.

Waiting rooms were reconfigured to accommodate social distancing guidelines. Patients were seated with spacing between them, reducing the capacity of waiting areas. One‐way systems were commonly used to guide the flow of people within the clinic spaces. Initially, conformity was high, although adherence decreased as the local and national guidance on COVID‐19 relaxed over time. Another approach was to guide students through different entrances and exits to manage flow, minimise interactions and maintain social distancing:

“We had social distancing in waiting rooms… [patients were] brought through to essentially wait in the [dental] chair that they were being treated in to try and reduce the volume of patients that were waiting in the waiting room… there was a one‐way system in operation… fire exits [were] upgraded in terms of their lighting and floor signage, so that people could go up onto a higher floor and then come back down onto a lower floor and go out of a sort of slightly different exit… one‐way traffic in and out.” [PARTICIPANT 7]

Coordinated clinic start times were implemented with patient bookings staggered and taken directly to the chairs rather than using the waiting areas to reduce patient numbers in any given space. While most measures were effective, there were minor challenges in managing student compliance. Continuous communication and emphasis on the importance of adhering to safety protocols were necessary to maintain compliance, especially among student groups. Where not proving inhibitory, one‐way systems and flow control have been retained at some schools.

### 4.3 Procedures and Equipment

Determining suitable alterations to procedures and equipment without disadvantaging students and patients was a major challenge. Many institutions introduced speed‐increasing electric handpieces, particularly in open‐plan clinics, with an aim to mitigate viral aerosol dissemination. Initially, operating speeds of 60 000 rpm were used based on the existing literature, with subsequent evidence demonstrating its efficacy [7, 12]. Retrofitting existing dental chairs allowed for the integration of advanced technology into the existing infrastructure:

“We changed almost entirely over to using jet spray electric motor‐driven handpieces… at the time, our estate was tired and in need of modernising. So, we had to retrofit a number of motors to dental chairs that didn't already have electric motors on them.” [PARTICIPANT 7]

Grants from organisations such as Health Education England and the NHS often funded purchases of speed‐increasing handpieces. The use of these handpieces underwent several shifts and considerations across the dental schools. There was hesitation and resistance among some staff to adopt electric handpieces:

“We involved [fifth year students] in some preclinical skills training, just in case they went into a VT practice that had a red band handpiece, to give them… a chance to use it… a lot of colleagues actually were concerned about the torque of them. And in someone who was slightly inexperienced… there could be a potential to create more iatrogenic damage than you would do with a high speed.” [PARTICIPANT 6]

“The disadvantages of… the speed increasing handpieces, are that nobody uses them [in primary care]. We don't have them widely available. There's a lot of problems with them at follow‐up at the moment, and although they're widely used in Europe, we felt that when our students were going out then to practice, they would be using things that they hadn't been trained on.” [PARTICIPANT 9]

Limited uptake was observed among vocational trainers, influencing the decision‐making process within the schools. Several schools extensively researched aerosol spread in turbines and micromotor handpieces, assessing different speed and airflow parameters. Speed limitations gradually increased as nascent evidence revealed minimal increases in viral aerosols at speeds up to 200 000 rpm, in the absence of “chip‐air”, airflow [8, 22]. Over time, some clinics reverted to using regular air turbines instead of electric handpieces. This transition was influenced by factors such as the differences in tactile sensation between handpieces, improved airflows and the need to align students' experiences with what they might encounter in general practice, where air turbines are predominantly used. Furthermore, the cutting efficiency of the electric handpieces at reduced speeds was diminished, so operative procedures took longer. While students are educated about different handpiece types, many schools have chosen to primarily utilise air turbines in their operative teaching and procedures.

“We still continued to have them on the units and a lot of students continued to use them. Obviously, there's limitations, it is quite slow for certain procedures. So as soon as we could start using the air rotors again, the students had the option of basically using either… but we have the option of reverting back because we've got the micromotors somewhere.” [PARTICIPANT 10]

In summary, there was an initial adoption of electric handpieces, which was followed by a partial shift back to regular air turbines due to practical considerations and the need to align student training with real‐world scenarios. Despite this, electric handpieces are still considered integral to the future of these clinics, with commitments to ongoing maintenance and incorporation into newly acquired dental chairs.

The significant investments in electric handpieces have been retained, if not always utilised. These provide schools with greater aerosol control and flexibility to adjust parameters based on procedural requirements or in the case of future challenges. An increase in teaching awareness of these tools has been reported, though some institutions are not implementing them on a regular basis due to their not reflecting real‐world primary care settings.

During the early stages of the pandemic, maximum patient clinic capacities were reduced, with appointments often staggered to maintain social distancing. As minimal research data was available at this time, extended fallow times were introduced to allow for adequate ventilation and reduce the risk of patient‐to‐patient aerosol transmission. The use of fallow time varied significantly across different clinics and institutions:

“In some rooms, the air change was non‐existent and it would be a 6 hour fallow time or 5 hour fallow time, and it was just impossible.” [PARTICIPANT 6]

For the majority, initial fallow times were set at arbitrary figures like 30 min, 45 min, or even up to an hour, primarily based on assumptions and a limited understanding of aerosol spread. These numbers were chosen to ensure safety but lacked a strong scientific basis.

“So people had arbitrary numbers of 30 minutes, 45 minutes or whatever. And then gradually research started to come through to show the times that will be suitable. But initially it was quite a high figure. It was anything up to an hour in between patients, and then gradually it reduced down to around 15 minutes… all based on the amount of air flow changes in your surgery, and what we realised at an early stage is that there was a huge difference between surgeries in the number of air changes per hour.” [PARTICIPANT 5]

To manage the fallow times effectively, timing systems were developed and mandatory training sessions implemented. Over time, the fallow period gradually reduced, and eventually, it was largely eliminated. In many instances, there is currently no fallow time between patients, indicating a return to pre‐pandemic operational procedures. The stringent measures that were once in place have been removed, reflecting the changing circumstances and evolving understanding of the situation.

There was a significant emphasis on the use of rubber dam in dental procedures, which was regarded as a crucial mitigating factor against the spread of bioaerosols. Dental staff and students were encouraged and, in many cases, mandated to use rubber dams during procedures.

“We try and get the students to use rubber dam most of the time anyway. So, for us working in a hospital setting with sort of gold standard for endo, obviously, and for composite work.” [PARTICIPANT 6]

This emphasis on rubber dams usage was consistent across various dental procedures and was a key part of the infection control protocols during the pandemic. The benefits of using rubber dam, such as cross‐infection control, improved visibility, and patient comfort, were recognised.

“We insisted that all procedures were done under rubber dam, which obviously is one of the great mitigating factors against the spread of aerosol. If you can separate saliva from the aerosol, then you know you're going a long way to preventing any spread.” [PARTICIPANT 5]

Nonetheless, while this was already routine in many circumstances, an increase was encouraged. There was a perceived reluctance in general practice to rubber dam use. Efforts were made within the educational context to encourage students to make rubber dam use more routine in order to remove this perception in the future.

“In [our Dental School], we encourage students to use rubber dam for every restorative procedure.” [PARTICIPANT 10]

The initial temporary suspension of using ultrasonic instruments lasted for a prolonged period, often forcing both instructors and students to rely on manual hand instrumentation. While this change was frustrating for both parties, it resulted in students significantly improving their skills with manual tools. The reintroduction of ultrasonic procedures in dental clinics was facilitated using local exhaust ventilation devices (LEV's) with HEPA filtration. These were put in place to address suspended aerosol particles. Nonetheless, the efficacy of these devices was unclear. In some instances, such as with ultrasonics, LEV devices remained after restrictions were relaxed.

Patient screening protocols were implemented at both pre‐appointment and on‐site stages. Patients were often contacted prior to an appointment to assess for COVID‐19 symptoms, or alternatively, assessed upon arrival.

“We used to have a screening procedure at the front of the hospital. So all patients would go through a COVID screening test. They would be asked about temperature and coughs and colds and smell and all that sort of thing before they came in. And obviously it was mandatory [for patients] wear masks throughout the clinic, apart from when receiving treatment.” [PARTICIPANT 5]

COVID‐19‐positive patients were segregated and only treated for urgent care needs. Masks were mandatory for patients at all points up to and after treatments. These extra measures proved an extra layer of safety to help minimise potential spread of respiratory droplets in shared spaces [23]. COVID‐19 screening information is still being captured, with clinical forms querying signs and symptoms of COVID‐19.

During the pandemic, there was meticulous adherence to PPE protocols. The PPE requirements were stringent and varied slightly. During AGPs, students and staff wore full PPE, including FFP3 masks, visors, gloves, gowns, and aprons, with the specifics determined by local policy. Some students had different face shapes, leading to the need for multiple types of masks. Fit‐testing for masks was conducted to ensure a proper and secure fit for both staff and students. Obtaining and sustaining a supply of appropriate masks for fit testing was a logistical challenge. Some masks were preferred initially but became harder to procure, leading to changes in the type of masks used. Some students also used powered respirator hoods due to issues with mask fitment, religious reasons or sensory impairments.

“Supply of different FFP3 masks changed at different times. I think a lot of people were fitted to some of the 3M masks at the start, which then became harder to get hold of, and we had to change and be refit tested. Some of our students elected to wear hoods either for religious reasons or because they didn't pass fit testing.” [PARTICIPANT 3]

In some instances, dental technicians were trained as fit testers:

“We trained up [those] dental technicians to be able to become fit testers and they then cascaded that down locally. So we were able to have in‐house fit testing for the staff, and then we also employed them as well to do the fit testing for the student cohorts as they came back into programme.” [PARTICIPANT 7]

As the pandemic progressed and restrictions eased, there was a shift towards individual responsibility, allowing clinicians and students to make decisions about the level of protection they deem necessary based on their circumstances and patient interactions. Post‐pandemic, increased awareness about the importance of PPE has generally led to more consistent usage compared to pre‐pandemic times. Much of the PPE has returned to pre‐pandemic levels, but there seems to be a greater awareness and appreciation for its use:

“Now I think people are a bit more strict and observant of PPE. And they're less likely to forget compared to what we used to be in the past.” [PARTICIPANT 2]

Nonetheless, some additional PPE has remained, such as the retention of single‐use aprons. In several scenarios, full face visors have been preferred to smaller protective glasses.

“So we're back to normal PPE I guess is what we call it… The visors we've kept actually, rather than little protective glasses. They're all still wearing a full visor… Mask. Just a routine, fluid‐resistant mask… And then they use just an apron, not a gown anymore.” [PARTICIPANT 1]

The use of respirator hoods, while crucial for safety, created communication challenges and contributed to patient anxiety. The barrier created by the hood hindered effective communication between patients and staff, especially supervisory staff working with students:

“There were problems with communication. So, being able to hear adequately and engage patients with conversation, and also members of staff… supervisory staff, if we're talking about students, for example, being able to effectively communicate between the sort of sound barrier that the hood created. It could at times be problematic.” [PARTICIPANT 7]

## 5 What Were the Barriers to the Pandemic Response?

### 5.1 Financial

Institutions had to make rapid decisions, balancing short‐term investments with long‐term planning. The consideration of financial implications was significant, especially when deciding on major changes such as improving ventilation systems or transitioning from open‐plan clinics to single‐chair surgeries.

“[We] wanted the building to have a 10‐unit ventilation. [We couldn't]. We wanted to be able to access different settings like outreach and increase that, and we couldn't. … some of those elements [were] difficult to implement because of either finances or because of physical restrictions.” [PARTICIPANT 2]

Modifications to create safer dental environments were often driven by available funding. Dental schools faced difficult choices, balancing the need for safety measures with the financial limitations they encountered. Some institutions, particularly hospitals with better financial positions, were able to invest in modifications to enhance air quailty and safety protocols. Others, with tighter budgets, found such improvements to be prohibitively expensive. The financial constraints also influenced decisions on staff employment, with funding required for additional personnel due to the need for continuous supervision in single‐room clinics.

“Finance. Because if we did reduce student activity, we need to increase the number of staff. And that is very, very costly. So, even without the additional consumables that are all round and the stuff attached to that, it's actually getting staff to come in more. And in the middle of any pandemic, there's more illness, there's more stress, children at home… so, you know, increasing that number of staff would be the biggest cost.” [PARTICIPANT 9]

The financial limitations impacted decisions related to various aspects, including transitioning from air turbines to electric handpieces, modifying existing clinics, and exploring options like partitioning areas with sliding doors. These challenges highlighted the complexities of implementing necessary changes while working within the financial constraints of the healthcare system.

“[Changing to electric handpieces was] obviously a massive undertaking with a number of units that we have on clinic, and it required an investment of well over half a million pounds to achieve that… The key [barrier] is financial, I'm afraid to say.” [PARTICIPANT 5]

Additionally, there were complexities in funding allocation, with financial support from governmental bodies, such as the Scottish Government, being directed to the NHS, which shared buildings with universities. This shared infrastructure created additional logistical challenges in financing and implementing necessary modifications.

There was also uncertainty about future funding for fit testing. The responsibility for providing fit testing and associated funding varies based on contracts and arrangements between dental hospitals, universities, and Health Education England (HEE). The funding sources and responsibilities might change, leading to a potential variation in fit testing protocols and mitigation potential across institutions [24, 25].

In summary, financial constraints played a central role in the decision‐making processes, shaping the strategies employed to improve ventilation and create safer dental spaces during the pandemic. These challenges highlight the need for careful planning and allocation of resources to ensure the safety of dental schools while managing budgetary limitations.

### 5.2 Estate Management

The implementation of safety measures, including enclosed units for AGPs and improvements in ventilation, faced significant challenges due to the physical limitations of existing buildings. Along with financial limitations, physical restrictions within existing estates hindered plans for creating contained areas with controlled airflows. Furthermore, the mechanics of clinics, including the placement of windows, gas supplies, and water sources, often limited the options for clinic adaptations to improve airflow or isolation procedures.

“We couldn't fit [as many pods] in. So, we just put them where we could fit them, and that caused some problems, especially with the early ones, because the bays that they were in were quite small anyway. The nurses found it difficult to manoeuvre when they were in the pod… and obviously [the pods] got quite warm.” [PARTICIPANT 6]

Ventilation upgrades were not always possible, with building layouts and shared NHS facilities making improvements challenging to implement. These challenges underscore the complexities faced in attempting to implement optimal safety measures within existing infrastructures.

“[Building updates were] difficult to implement because of either finances or because of physical restrictions as it's in a different building or a completely different setting.” [PARTICIPANT 2]

### 5.3 Applying Guideline Application and New Research

When considering decisions as to various options for improved ventilation, whether through air purifiers, filters, or mechanical ventilation, schools adhered to collective wisdom and the existing research available. Where possible, measures were adapted as new information arose. Nonetheless, the development of a national position on handling the challenges brought by the pandemic was a gradual process, marked by delays and uncertainties, with guidance varying between NHS trusts resulting in discrepancies.

“It [was] the NHS partner who [was] accountable for the changes through infection prevention and control. So the university… if we'd been an independent organisation, we probably would have moved in a different way.” [PARTICIPANT 5]

The interpretation of guidance and research varied significantly across different institutions. For instance, there were disparities in the acceptance and use of specific masks, such as FFP3 masks, with some health boards approving their use while others did not [26], despite the availability of these masks. This was particularly challenging for institutions working with multiple health boards:

“We work with [several] different health boards and the level of PPE was slightly different for each. Some would make you wear overshoes, hats, 2 pairs of gloves, 2 aprons, gowns, visors, FFP3 masks, et cetera. So, the process of getting hundreds of students face fitted for masks and finding masks and the sustainable source of masks was a big logistical issue, as I'm sure it was the same in every dental school. So, that was a big challenge.” [PARTICIPANT 6]

Additionally, there were challenges in evaluating the effectiveness of safety measures, especially concerning live viruses. The majority of research focused on water droplets and aerosol components [9, 11, 12, 22], not necessarily capturing the behaviour of the live virus. Moreover, adapting safety measures to older buildings with limited air exchange capabilities posed a significant challenge. While external research was considered, the primary focus was on understanding and implementing measures that were feasible and effective within the local context and constraints of the existing infrastructure. Though discussed to a lesser extent, some institutions preferred awaiting formal guidance about changes, citing the volume, and at times contradictory nature, of research early in the pandemic as prohibitive:

“We followed guidance rather than research… Because when you're making these decisions about what you do as a unit… you can read papers here and there. But obviously all the research was quite new and it was coming out thick and fast and some of it was quite contradictory as well… So it wasn't until the guidance came through, which sort of categorically [changed our practices].” [PARTICIPANT 10]

The lack of precise information, especially regarding fallow times and handpiece use, led to decisions being made with limited data. In some instances, decisions were influenced by the availability of resources and infrastructure, such as the ability to conduct AGPs and acquire electric handpieces. Experts in decontamination and infection control were consulted, although opinions varied, reflecting the uncertainty and evolving nature of the situation.

The understanding of aerosols, their spread, and their potential to carry viruses remained uncertain, highlighting the need for extensive studies to comprehensively explore aerosol behaviours, viral carriage within aerosols, droplet size, distances travelled, and infectivity levels. At the beginning of the pandemic, the existing knowledge was limited, leading to uncertainties in the protocols and safety measures implemented. Questions were raised about the basis for certain guidelines, such as the requirement for 10 ACH, emphasising the lack of comprehensive understanding and the need for further research in this area.

“I think the big thing is, we still don't really know an awful lot about aerosols and how far they're spread and do they spread and do they carry virus… those are the studies that need to be done… is that we actually learn more about aerosols and viral carriage in aerosols and droplet size and droplets distance… and infectivity sort of levels… We may have got it all wrong. We just assumed a heck of a lot… Even things like the 10 air changes per hour… [it] was this magic number you had to reach and… what was that based on?” [PARTICIPANT 1]

The absence of established protocols for supervising and addressing these uncertainties highlighted the challenges faced in navigating the unknowns surrounding aerosol transmission and safety protocols.

### 5.4 Managing Fear

Fear played a significant role in decision‐making processes during the pandemic. The fear factor, driven by genuine concerns for safety, influenced various aspects of managing the crisis, including implementing protocols, convincing staff, and supervising students.

“There [was] a lot of staff that were very resistant to the risk, obviously, and that was… it was managing the risk of what's your chances of an aerosol containing COVID and infecting a member of staff.” [PARTICIPANT 6]

Overcoming this fear required clear communication, expert input, and efforts to manage risks effectively. The challenges posed by fear underscore the importance of providing accurate information and ensuring that decisions are based on a balance between safety and the necessity to continue essential operations.

“Everyone was lashing about in the dark trying to get it right, and if in doubt it was shut it down.” [PARTICIPANT 1]

### 5.5 Reflections on the Future‐Proofing of Dental Schools

Although not all UK dental schools contributed their experiences, a substantial majority participation offers valuable insight into the pandemic response. Whilst many researchers have delved into this subject through the use of questionnaires [15, 16, 17, 27], this study utilised interviews to gain a deeper understanding of institutional changes. The lack of a unified national response to the pandemic led to varying approaches and experiences across dental schools, resulting in a sense of unfairness among students. The absence of a centralised strategy impacted the speed of educational adaptations for students, with dentistry being particularly affected among higher education disciplines.

“It was quite tricky to then adapt to the circumstances in a dental school. So what would we do differently? I think it's probably more kind of national bodies realising that dental schools were different… and funding it very quick.” [PARTICIPANT 4]

Despite challenges, dental schools demonstrated resilience and innovation during the pandemic. Implementing single surgeries for undergraduates, although increasing supervision burdens, allowed for more personalised attention and contributed to the overall positive experience of the students. The pandemic highlighted the importance of adaptability, and the use of pods and enhanced ventilation in some institutions improved safety measures. However, reliance on specific setups poses challenges if future viral threats necessitate similar adjustments. As the situation continually evolves, there is a need to prepare for potential future disruptions while learning from the experiences and adaptations made during the pandemic.

The general perspective was that the concept of open‐plan clinics is not conducive to aerosol containment. While the idea of creating more single‐bay surgeries is appealing from an infection control perspective, the feasibility of this approach is challenged by the need for additional supervisory staff and the limitations of existing buildings. Despite these challenges, dental schools are actively seeking innovative solutions to strike a balance between infection control and practicality, recognising the evolving landscape of healthcare challenges.

The uncertainty and lack of understanding about the virus were highlighted. Many decisions were made in the absence of clear knowledge and guidelines, leading to a cautious approach of shutting operations down as a precautionary measure.

“When I look back and think about what we did and what other schools did, it was whatever was the local collective wisdom that was felt to be consistent with safety for both patients and staff and visitors. But there was no rationale or clear underpinning science for a lot of the changes that were made.” [PARTICIPANT 5]

Moving forward, there's a strong emphasis on being informed and knowledgeable to make better decisions, ensuring that responses are based on understanding rather than ignorance. This learning is vital for future preparedness, emphasising the importance of having clear guidelines and protocols in place to navigate similar situations more effectively.

“We would be in a much better place if we had another pandemic tomorrow because we've still got that infrastructure there from before and a bit of know‐how about how to work in that environment now.” [PARTICIPANT 6]

There is a growing awareness of the critical role ventilation plays in infection control. In future clinical space designs, prioritising proper ventilation systems, alongside other considerations like equipment choices, is crucial. Electric handpieces are recommended due to their lower aerosol generation potential, and the layout of clinical spaces is being re‐evaluated. Spaces with more room and traditional layouts proved easier to adapt during the pandemic, suggesting that future designs might benefit from spacious, well‐ventilated, and flexible layouts.

The lessons learned from managing the pandemic in dental education settings emphasise the need for clear guidelines, timely decision‐making processes, and a balance between short‐term emergency responses and long‐term planning. A common set of loose guidelines could provide flexibility while ensuring safety for students, patients, and staff, preventing future disruptions in education.

It's positive to note that despite the challenges and adaptations made during the pandemic, many dental schools did not experience outbreaks of viruses within the clinical environment. Additionally, although there was some hesitancy among some student cohorts regarding vaccination uptake [28], the impact of vaccinations appears to have played a significant role in preventing staff‐related issues and clusters of cases. This indicates the effectiveness of vaccination efforts in maintaining a safer environment.

The implementation of electric handpieces and the anticipation of effective vaccines have significantly shaped schools' approaches during the pandemic. In many circumstances, the decision to invest in proven technologies like micromotors rather than extensive structural changes indicates a pragmatic approach based on established evidence. This decision‐making process reflects the adaptability and focus on evidence‐based strategies, ensuring a balance between safety and operational continuity.

It must be emphasised that, although many UK dental schools may have responded in similar fashions to specific challenges, for example, the use of HEPA filters, the lack of evidence base behind some of the decision‐making must not automatically lead to this being the consensus approach. Further research investment is essential to provide robust building blocks for future decision‐making.

## 6 Conclusion

British dental educational institutions demonstrated proactive measures and innovative approaches in safeguarding their students from irreversible educational setbacks amidst the shifting complexities generated by the COVID‐19 pandemic. Devising a research‐data‐supported plan to tackle future challenges would enable a more timely, unified response, ameliorating the impacts on student education and further developing resilience in the dental school system. Given that many dental clinics worldwide share similar setups, particularly in open‐teaching environments, the strategies implemented in the UK are likely translatable to a global context. International audiences can derive significant value from these insights by identifying applicable elements for their specific contexts, which could strengthen the global preparedness of dental education systems for future crises.

## Author Contributions

J.J.V., K.V.C. and J.C. contributed to the conception, design, acquisition and analysis of data, drafting and revising the manuscript. D.J.W. contributed to conception, design, and manuscript revision. G.E. contributed to data acquisition and manuscript revision. P.E.L. and B.R.N. contributed to conception, design and manuscript revision.

## Ethics Statement

Ethical approval was acquired through the University of Leeds Dental Research Ethics Committee (reference: 080323/JV/369).

## Conflicts of Interest

The authors declare no conflicts of interest.

## Data Availability

Research data are not shared.
